# Comparison of echocardiography, magnetic resonance imaging and histopathology for the imaging evaluation of intracardiac masess

**DOI:** 10.1186/1532-429X-14-S1-P295

**Published:** 2012-02-01

**Authors:** Juan M Bonelli, Eric Kimura, Gabriela Meléndez, Francisco Castillo, Sergio G Olmos, Nelsy C González, Erick Alexánderson, Luis Marroquin, Fernando Iñarra, José E Telich-Tarriba, Aloha Meave

**Affiliations:** 1 Cardiovascular Magnetic Resonance Imaging, National Institute of Cardiology "Ignacio Chávez", Mexico City, Mexico; 2 Cardiovascular Computed Tomography, National Institute of Cardiology "Ignacio Chávez", Mexico City, Mexico; 3 Nuclear Cardiology, National Institute of Cardiology "Ignacio Chávez", Mexico City, Mexico

## Background

Cardiac Magnetic Resonance (CMR) has demonstrated being an ideal method in the diagnosis of cardiac masses because of its accuracy in tissue characterization. The main objective was to compare the utility of CMR in the diagnosis of cardiac masses with Echocardiography (Echo) having the histopathology findings as the gold standard.

## Methods

Thirty four patients were enrolled: twenty two males, twelve females, age between 15 days and 80 years old (mean 38 years old) with diagnosis of cardiac mass underwent echo and CMR before biopsy or surgery. Echo gradient cine images in multiple views, T1 and T2 weighted sequences, and additional information derived from first pass perfusion imaging and inversion recovery post gadolinium delayed images allowed an accurate diagnosis in the majority of cases.

## Results

Table 1 summarizes the main results. MRI was able to diagnose correctly 26 of 34 cases (76.5%) while echo only diagnosed correctly 11 of 34 cases (32.3%).

**Table 1:** Results

Masses	Echocardiogram diagnosis	Cardiovascular MRI diagnosis	Histopathology diagnosis
Thrombus	4 (2/8)	7 (7/8)	10
Myxoma	8 (7/10)	9 (9/10)	10
Sarcoma	-	5 (2/2)	2
Endomyocardial fibrosis	--	3 (2/2)	2
Rhabdomyoma	3 (1/2)	3 (2/2)	2
Pericardial mass	2	2	--
Other	1 (1/5)	3 (2/5)	5
Papillary fibroelastoma	--	1 (1/1)	1
Metastasis	--	1 (1/2)	2
Non-specific	16	--	--
Non-thrombus / non-masses	--	--	2
Total	34	34	34

## Conclusions

We conclude that CMR is an advantageous tool over echo in detection, and complete morphological and functional evaluation as hemodynamic repercussion of cardiac masses. This research also demonstrated CMR capacity to exclude or confirm the presence of a cardiac mass when the echo was equivocal. CMR predicts the likely diagnosis of the tumor in the majority of the cases. A comprehensive imaging protocol is essential for accurate diagnosis. However, histopathology diagnosis remains the gold standard, and in some cases malignancy cannot be definitively excluded on the basis of CMR images alone.

## Funding

National Institute of Cardiology "Ignacio Chávez", Mexico City, Mexico.

